# Can We Push the “Quasi-Perfect Artifact Rejection” Even Closer to Perfection?

**DOI:** 10.3389/fninf.2020.597079

**Published:** 2021-01-14

**Authors:** Makoto Miyakoshi, Lauren M. Schmitt, Craig A. Erickson, John A. Sweeney, Ernest V. Pedapati

**Affiliations:** ^1^Swartz Center for Computational Neuroscience, Institute for Neural Computation, University of California San Diego, La Jolla, CA, United States; ^2^Developmental and Behavioral Pediatrics, Cincinnati Children's Hospital Medical Center, Cincinnati, OH, United States; ^3^Department of Pediatrics, University of Cincinnati College of Medicine, Cincinnati, OH, United States; ^4^Divisions of Child and Adolescent Psychiatry, Cincinnati Children's Hospital Medical Center, Cincinnati, OH, United States; ^5^Department of Psychiatry, University of Cincinnati College of Medicine, Cincinnati, OH, United States; ^6^Divisions of Neurology, Cincinnati Children's Hospital Medical Center, Cincinnati, OH, United States

**Keywords:** line noise artifact, EEG, MEG, spatial filter, temporal filter

## Introduction

Removing line noise from data is a basic need in the field of electrophysiological studies. Using a notch filter, which is a narrow band-stop filter to suppress the line noise frequency centered at 50 or 60 Hz, is still a widely accepted practice. However, for certain applications notch filter is not a preferred choice. For example, for Granger causal analyses, making a deep “hole” on the background PSD makes the empirically estimated VAR model order increased, indicating unstable model and poor estimate of parameters (Barnett and Seth, 2011). To avoid this problem, line noise rejection without affecting the background PSD is required. In this paper, we will focus two alternative solutions to notch filter, namely ZapLine and CleanLine, and the suggested practice to get the most out of them and the underlying complementary mechanism.

The “ZapLine” algorithm (de Cheveigné, 2020) is a novel and promising approach to remove line noise artifacts. Described by the authors as “quasi-perfect artifact rejection,” the paper demonstrated impressive results for this ubiquitous concern in electrophysiology. In this short commentary, we validate the technique and suggest optimizations to further improve performance.

The unique assumption of ZapLine holds there should be no perfect solution in either the spatial domain or spectral domain alone, but the best solution to separates line noise from brain source activity, exists in spatio-spectral domains. To roughly describe the approach, (1) ZapLine first separates the original data into two filtered signals: a narrow band-stopped (i.e., notch filter) signal and a band-pass filter that captures the noise artifact (original_data = band-stopped_data + filtered_artifact); (2) ZapLine fits a spatial filter to the filtered_artifact that is optimized to capture the maximal variance of the extracted line noise; (3) ZapLine accepts user-input parameters that determines the number of dominant artifact subspaces for rejection; (4) ZapLine adds back the cleaned filtered_artifact to the band-stopped data. Thus, the background power frequency spectra within the line-noise frequency bands is recovered. In our hands, we noticed that using a time-domain solution (CleanLine) in post processing may further improve performance. By design, ZapLine does not account for temporal non-stationarity as it depends on a spatial filtering. An assumption of spatial filtering is that relations among a fixed set of scalp electrodes should stay unchanged throughout the recording. However, in real world data this assumption is often violated. In fact, we recently demonstrated the significant consequences of violating non-stationarity with another spatial filter method, independent component analysis (ICA) (Hsu et al., 2018).

CleanLine (Mullen, 2012) is an example of line-noise removal algorithm that principally works in the time domain. This solution is based on the original idea that multi-taper decomposition can be used to identify and remove line noise components while minimizing background signal distortion (Mitra and Pesaran, 1999; Mitra and Bokil, 2007). The Cleanline algorithm, uses a short sliding window (default 4 s with 1-s step size) to generate a frequency-domain regression model to estimate the amplitude and phase of a deterministic sinusoid of a specified frequency. The fitted signal representing line-noise is subtracted from the data. We can see several interesting contrasts between the two algorithms: ZapLine uses a stationary spatial filter and as such, multivariate, while CleanLine uses a non-stationary temporal filter that is applied univariately. Thus, we predicted that CleanLine when used in post-processing of ZapLine-cleaned data should improve line-noise cleaning as the two algorithms are complementary in nature.

To test our prediction, we chose a clinical dataset exemplar that contained a very high level of line-noise artifact (60 Hz in US) as a challenging example of real-world data preprocessing. If we could observe improvement in the result from applying “quasi-perfect” ZapLine by using CleanLine, it provides evidence that ZapLine results still leaves space to be improved by methods with complementary nature. In performing this comparison, reduction of the line-noise power is one critical measure of performance. On the other hand, the influence of the filter on off-target frequency range is another critical measure, as it indicates undesired “side effect.” To evaluate it, the test data low-pass filtered at 55 Hz was used to serve as the ground-truth. Any deviation from the 55-Hz low-pass filtered data indicates the “side effect.”

## Methods and Results

### Data Used

The data contained 5-min of resting-state recording from a 128-channel recording system sampled at 1,000 Hz (NetAmp 400 and Hydrocell electrode nets, Phillips/EGI, Eugene, OR) recorded in a project of National Institutes of Health U54 Fragile X Center.

### Data Preprocessing

The data were imported to EEGLAB (Delorme and Makeig, 2004) running under Matlab 2017b (Mathworks, Natic, MA). High-pass filter (FIR, Hamming window, cutoff frequency 0.5 Hz, transition bandwidth 1 Hz) was applied to the data to remove near-DC component. From the beginning and the ending of the data, 10-s windows were removed to avoid possible irregular artifact that is typically present due to recording-related artifacts.

### Parameters for Zapline and Cleanline

There were five datasets prepared from the same data processed differently. The first data was Raw, which is just high-pass filtered and edge-trimmed, as described above. This data served as a basis for any of subsequent filter applications. The second data was 60-Hz low-pass filtered, which served as ground-truth of the intact off-target frequency range data, namely below 60 Hz. The third data was ZapLine-processed. The fourth data was CleanLine-processed. Finally, the fifth data was ZapLine-processed then CleanLine-processed. The parameters used were as follows.

The 60-Hz low-pass filter was applied using *pop_firws*() function (FIR, Blackman window, cutoff frequency 55 Hz, transition bandwidth 5 Hz). This filter was a part of an EEGLAB plugin firfilt (ver. 1.6.2) written by Andreas Widmann. Because the line noise was known to have an extraordinary level of power, Blackman window (−74 dB) was selected instead of EEGLAB default of Hamming (−53 dB) to make sure the suppression is sufficient. For the same reason, the low-pass fitler was designed so that maximum suppression was achieved at 57.5 Hz to secure a margin against the 60-Hz artifact.

ZapLine (version 18-Feb 2020, generated by *nt_version* that is a Matlab function to display ZapLine version) was applied with the following parameters: Target frequency, 60 Hz (and auto-selected harmonics up to 500 Hz); Size of FFT, 1024; Number of components to remove, 25, which was determined by fitting two lines to determine a bisection point that minimizes the sum of errors for the two fits (Dmitry, 2012).

CleanLine was applied using Matlab function cleanLineNoise() included in PREP Pipeline 0.55.3 (Bigdely-Shamlo et al., 2015) was used. This PREP-implemented version of CleanLine, labeled as cleanLineNoise(), received a critical bug fix since the original CleanLine had released. We used the following parameters: Target frequency, 60 Hz and its integer harmonics up to 500 Hz; fscanbandwidth, 2; taperbandwidth, 2; taperwindowsize, 4; taperwindowstep, 1; tau, 100; pad, 2; fpassband, [0 500]; maximumiterations, 10.

### Results

**Figure 1:**
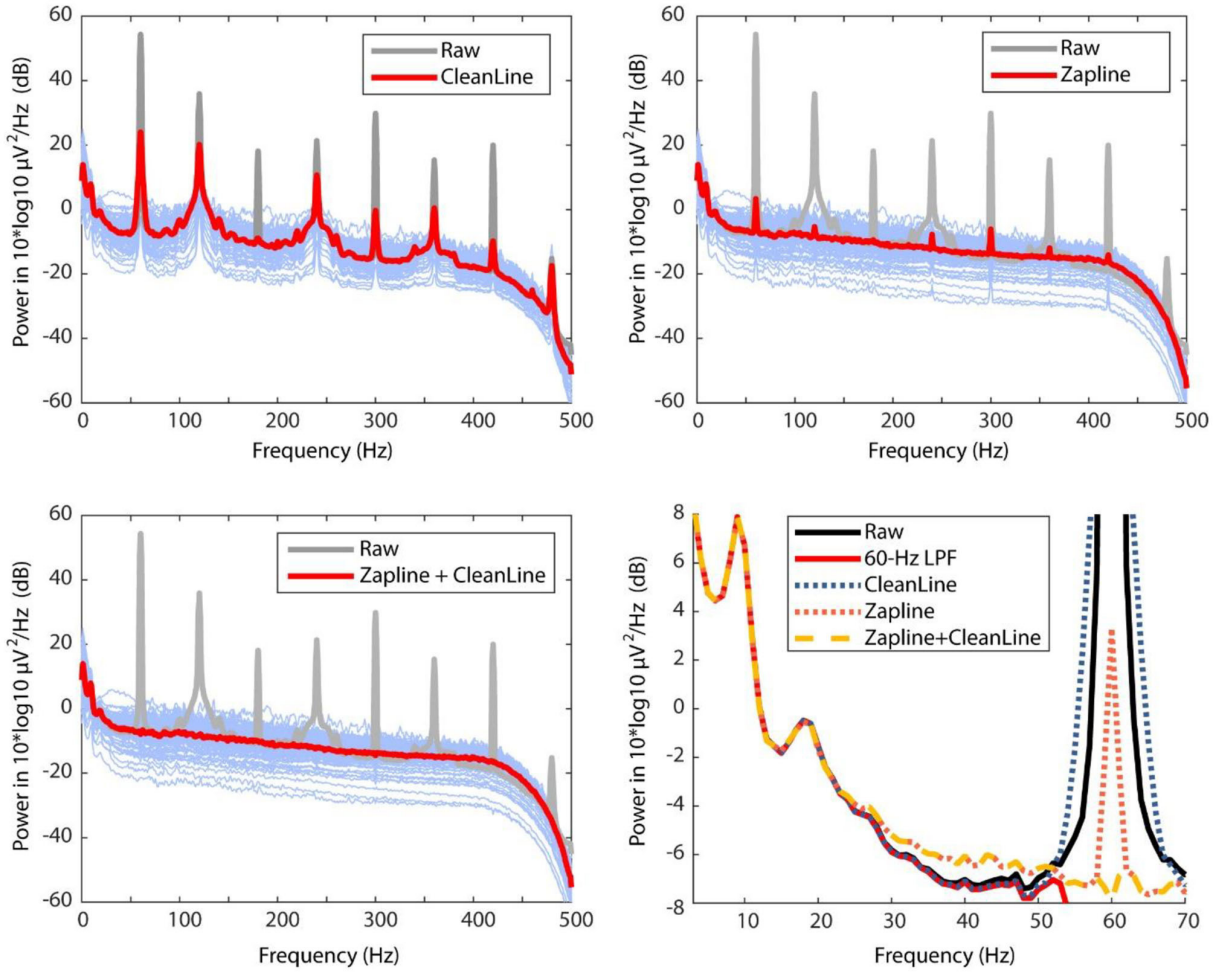
Comparing power spectral densities (PSD) across raw data (gray), CleanLine result (top left), ZapLine result (top right), and ZapLine followed by CleanLine (bottom left). The thicker lines indicate average PSD across all the 128 electrodes, and the thin blue lines represent cleaned PSD of each electrode. To evaluate influence on the non-target frequency bands, PSD under 70-Hz range is magnified with overlaying PSDs of different conditions (bottom right).

A power spectral density (PSD) comparison demonstrates ZapLine's performance was better than CleanLine, but further improved when CleanLine was used successively after ZapLine (Figure 1). The result confirmed that our prediction was correct. Next, we investigated if these processes influenced non-target frequency bands below 60 Hz (Figure 1, bottom right). ZapLine deviated from ground-truth (low-pass filtered) PSD inflating power above 25 Hz which was not seen in the CleanLine results. The result can be explained as ZapLine implements a spatial filter that is not strictly limited to certain frequency bands but aims to minimize the influence on non-targeted frequency bands. In addition, the large number of components (25/128) rejected may have exacerbated this effect. By reducing the number of components to reject, the impact on non-targeted frequency bands should be reduced, but at the cost of less line noise suppression. In contrast, CleanLine method (explicitly limited to address 60 Hz (and its multiple integers) +/– 2 Hz) did not show any deviation in the non-targeted frequency band.

**Figure 2:**
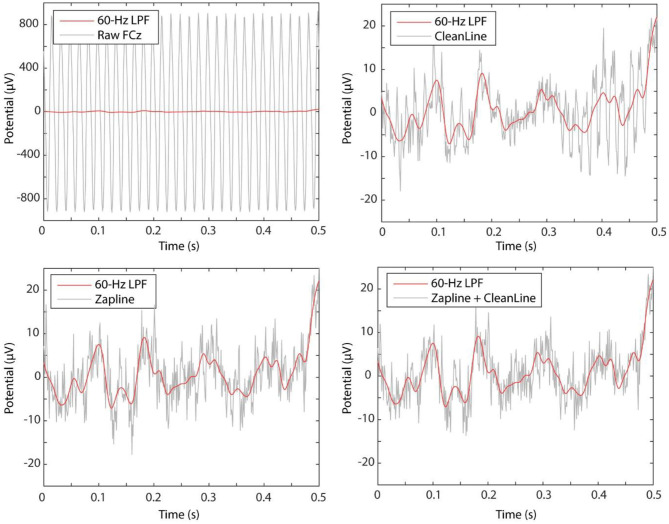
Comparing the first 3 s of the data at FCz. The raw data showed +/– 900 μV range of data (top left), which was brought down to a more standard range of +/– 40 μV by both CleanLine (top right) and ZapLine (bottom left).

Visual inspection of the time-domain data shows that both ZapLine and CleanLine reduced the very high (over +/– 900 μV) raw data amplitude to a standard EEG amplitude range (+/– 40 μV) (Figure 2). It is apparent in the ZapLine tracing, that the algorithm was more effective at power-line noise suppression than CleanLine. Unlike the spectrogram, the visual impression in the raw tracing of the advantage of ZapLine + CleanLine over ZapLine alone is subtle (Figure 2, bottom right).

## Discussion

In this study, we made a prediction that “quasi-perfect” artifact rejection performance by ZapLine may be further improved by using a method with a complementary properties that were equipped by CleanLine. The result in the PSD plots supported our prediction, and demonstrated that CleanLine used as a post-process of ZapLine indeed improved the line noise removal. The explanation for the result is that CleanLine's sliding-window based non-stationary approach complements ZapLine's stationary spatial filter approach. The result indicates that whenever one uses ZapLine, CleanLine may be used together as a post processing to easily outperform the performance of ZapLine alone. Thus, our theoretical prediction was confirmed by using empirical data, which makes an immediately useful suggestion for practice of line noise artifact rejection in the field of electrophysiology.

In addition to the performance of line-noise power suppression, there is another important aspect that needs to be considered, which is the influence on off-target frequency range i.e., below 60 Hz in the current case. In this extreme line-noise example in which the raw data looks like +/– 900 μV sine wave (Figure 2), the aggressive line noise removal using ZapLine (25/128 components removal, suggested by elbow detection algorithm) introduced unwanted off-target frequency range distortion between 30 and 50 Hz. To be fair, this particular test was akin to a “torture test” using an extreme data example. As such, the result may not generalize to other datasets, so caution needs to be taken. However, the result is still informative to learn the critical property of ZapLine: it does not hard-limit a frequency range to work on. On this point, ZapLine is different from notch filter or CleanLine. This property of ZapLine derives from the process of using a spatial filter to *maximally* capture the target frequency power; if it were a spatial filter that captures *only* the target frequency power, would it not cause the undesired distortion in the off-target frequency ranges. However, designing such a spatial filter is impossible because a spatial filter cannot not have explicit control over frequency domain. This off-target effect should occur in application to more normally-looking data even if the effect is in much lower level, and it is difficult to predict its behavior since it depends on both signal characteristics and user parameters. Based on these observations, we recommend that in using ZapLine in general, it is a good practice to evaluate not only the reduction of the line noise power but also power below the line noise frequency, particularly if large amounts of line noise and/or a large number of components are removed. It is also a good idea to watch the number of components removed across subjects. If a subject show outlier values, one might make sure whether the off-target frequency still remains minimally impacted.

Related to the argument above, we propose use of some objective criterion to the optimum number of components for rejection in addition to visual examination using analyst's expert knowledge. Needs for processing large EEG database has been surging recently. To be adopted to a part of fully automated preprocessing, ZapLine primarily needs to have an additional solution to determine the number of components for rejection. We believe that the use of bisection point method we demonstrated in the current study could be one such an approach to determine the optimum number. The detection of outlier can be done after preprocessing all the data sets in a data base by generating a distribution of the number of components to be removed. If outliers are found, it suggested these datasets needed to spend abnormally large number of components compared with other datasets, indicating problematically large line noise contamination in these datasets. Such a distribution would provide a data-driven, empirical criterion to separate abnormal from normal, which would be also useful for other studies.

The current results warrant further investigation on ZapLine's impact on phase integrity, such as phase coherence across multiple electrodes. Despite these considerations, we are enthusiastic about the development and optimization line noise removal techniques which broadly improve data retention in circumstances in which optimal recording conditions may not be feasible or frequencies within line-noise frequencies may be of interest.

## Author Contributions

MM: conceptualization, methodology, software, validation, formal analysis, and investigation, writing—original draft preparation, and visualization. LS, CE, JS, and EP: resources and data curation. EP: writing—review and editing, project administration, and funding acquisition. MM and EP: supervision. All authors have read and agreed to the published version of the manuscript.

## Conflict of Interest

MM, EP, JS, LS, and CE work on NIH funded studies in which development of EEG biomarkers are a central aim. MM owns a company GYREE (San Diego, CA). EP has received past research support or consulting from American Academy of Child and Adolescent Psychiatry, Proctor & Gamble, Eccrine Systems, and Autism Speaks. CE has received funding from Confluence Pharmaceuticals, Novartis, F. Hoffmann-La Roche Ltd., Seaside Therapeutics, Riovant Sciences, Inc., Fulcrum Therapeutics, Neuren Pharmaceuticals Ltd., Alcobra Pharmaceuticals, Neurotrope, Zynerba Pharmaceuticals, Inc., and Ovid Therapeutics Inc. to consult on trial design or development strategies and/or conduct clinical trials in neurodevelopmental disorders. LS consults for Confluence Pharmaceuticals. JS consults to VeraSci and has received support from NIH and Sichuan University.
